# Productivity change of national health systems in the WHO Eastern Mediterranean region: application of DEA-based Malmquist productivity index

**DOI:** 10.1186/s41256-018-0077-8

**Published:** 2018-08-01

**Authors:** Maysoun Dimachkie Masri, Eyob Zere Asbu

**Affiliations:** Health System Financing Division, Department of Health, Abu Dhabi, United Arab Emirates

**Keywords:** Data envelopment analysis (DEA), Efficiency, Eastern Mediterranean region of the World Health Organization (EMR), Output-oriented Malmquist productivity index (MTFP), Productivity, Productivity change, Technical efficiency, Total factor productivity (TFP) change

## Abstract

**Background:**

The pursuit of efficiency and productivity is one of the goals of health systems. In the era of Sustainable Development Goals and particularly the move towards universal health coverage, it is imperative to curb wastage of resources to ensure sustainable access of the population to needed and effective health services without enduring financial hardship. This study aims to assess total factor productivity change of national health systems of 20 countries in the WHO’s Eastern Mediterranean Region.

**Methods:**

Data Envelopment Analysis (DEA)-based Malmquist index is used to assess total factor productivity change and its components – efficiency change and technical change. To assess the robustness of the Malmquist index estimates, bootstrapping was performed. Outputs used are life expectancy at birth for both sexes and infant mortality; while total expenditure on health per capita in international dollars (PPP) is used as a measure of input. Panel data for the period 2003–2014 was extracted from databases of the WHO and the World Bank.

**Results:**

In all but five countries covered in the study, a decline in the mean total factor productivity is observed during the period 2003–2014. The decline is driven by technical regress. In all countries, the technical change component of the Malmquist TFP index is less than unity (range: 0.896 to 0.945). All countries exhibited growth in efficiency (efficiency change exceeding one) except two countries (Djibouti and Iraq). The growth in efficiency was mainly due to change in scale efficiency. Overall, total factor productivity in the region declined by 3.8%. This was due to a 9.1% decline in technical change, which overshadowed the 5.8% increase in efficiency. Three countries - Libya, Qatar and Yemen – showed a marginal growth in total factor productivity. There was no change in total factor productivity in Kuwait and Lebanon.

**Conclusion:**

The decline in total factor productivity over the study period is likely to hamper achieving the targets of Sustainable Development Goal 3 of ensuring healthy lives and promoting well-being for all at all ages. It is recommended that country-level studies on efficiency and productivity of health systems be conducted in order to intensively examine the determinants of inefficiency and productivity decline and implement appropriate interventions that could enhance efficiency and productivity.

**Electronic supplementary material:**

The online version of this article (10.1186/s41256-018-0077-8) contains supplementary material, which is available to authorized users.

## Background

The healthcare system constitutes a small part of the economies of most countries in the Eastern Mediterranean Region (EMR) with the total expenditures on health (TEH) as share of gross domestic product (GDP) being much lower than global averages [1]. The region is faced with high burden of communicable and non-communicable diseases (NCDs). NCDs range from cardiovascular diseases, mental and behavioral disorders, diabetes mellitus, and malignant neoplasms [1–3].

The WHO estimates that 20–40% of total health spending globally is wasted [4]. The negative impact of wastage of this magnitude on the fiscal space for health to move towards a sustainable universal health coverage cannot be overemphasized. Improving the efficiency of healthcare spending implying the maximization of outputs for a given level of input using each country’s constrained available resources for healthcare is critical [1]. Current research suggests that there are considerable efficiency gains yet to be made by some countries in the region and major healthcare reforms are underway [5–8].

The assessment and comparison of productivity of national health systems entails a better understanding of a health system and its boundaries, goals, structure and other factors that affect its performance.

Health systems are defined in various ways. Multiple conceptual frameworks have been proposed to date to describe health systems [9]. As defined by the World Health Organization (WHO), a health system consists of all organizations, people and actions whose primary intent is to promote, restore or maintain health [10]. The overall goals of health systems include: (i) improving health and health equity; (ii) responding to people’s expectations; (iii) providing financial protection against the cost of ill-health; and (iv) making the most efficient use of available health resources [11].

The WHO’s framework clearly indicates that the pursuit of efficiency and productivity is a central goal of health systems, albeit a difficult one [12]. In the era of the Sustainable Development Goals and particularly the move towards universal health coverage (SDG) as stipulated in SDG 3, Target 3.8 [13], it is imperative to curb wastage of scarce health care resources to ensure sustainable access of the population to quality needed health services without enduring financial hardship. A decline in productivity as a result of inefficiency and technical regress is of serious concern to policymakers for it may [14]:

i. reduce the health gains to patients as a result of compromising quality of services;
ii. deny treatment to other patients who could have otherwise benefitted from the system had wastage been minimized;
iii. result in reduction in consumption in other sectors such as education, social services etc. as a result of the opportunity cost involved; and
iv. reduce society’s willingness to fund health services thereby creating a chronic problem of resource availability to the sector.

Amidst a burgeoning literature on frontier analysis of efficiency and productivity of health facilities, especially hospitals; there is limited information on current cross-country comparison of national health systems specifically in the EMR [5, 15, 19, 20]. Given the scarcity of cross-country health systems performance research in this region [5], this study uses the most recent publically available data to analyze productivity change of national health systems over 12 years. The challenge of prior efficiency studies of national health systems [19] was to find a way of measuring health system performance in a systematic way, to allow comparison across countries and over time. Despite criticisms leveled against it [20], the World Health Report 2000 has been one of the influential documents in assessing performance of 191 member states along the level and distribution of multiple indicators. One of the indices of performance, the level of health, ranked countries on how efficiently their health systems translate health expenditure (measured by health expenditure per capita in international dollars) into health as measured by disability-adjusted life expectancy (DALE). Out of the 20 countries of the Eastern Mediterranean region of the World Health Organization (EMR) included in this study, 4 were ranked among the 20 best performers out of 191 countries on the level of health (DALE) performance. These included: Oman (1st), Saudi Arabia (10th), United Arab Emirates (16th) and Morocco (17th). On the other hand Djibouti (163rd) and Afghanistan (150th) were among the low-performing countries. Since the WHO report, several research examined the efficiency of national health systems whether on a global level [15–20], or on a regional level [21, 22]. A study of OECD countries has shown that keeping health expenditure constant, life expectancy at birth could increase by 2 years if all countries would perform as efficient as the best performers. In contrast, with the level of inefficiency prevailing, a 10% increase in health spending will only increase life expectancy by 3 to 4 months [21]. In a study of the productivity of 53 national health systems of continental African countries [22], it was found that all health systems registered improvement in total factor productivity attributable mainly to technical progress.

In its simplest form, productivity is a ratio of health system inputs used to value health system outputs that the health system inputs create. Productivity change over time, which may be in the form of growth, decline or stagnation, results from efficiency change and technical or technological change or a combination of both. Analysis of temporal changes in productivity should thus focus on the drivers behind changes in efficiency and changes in technology.

In this study, we aim to estimate the total factor productivity (TFP) change of national health systems in 20 countries of the WHO Eastern Mediterranean Region using DEA-based Malmquist productivity Index [23]. The specific objectives of the study are to:

i. assess total factor productivity (TFP) change over the years 2003–2014; and
ii. estimate the contributions of efficiency change and technical (technological) change to TFP change.

Thus, this cross-country comparative study analyses the productivity change of each country, using an output-based DEA looking at by how much can output quantities be proportionally expanded without altering the current input quantities used.

### Eastern Mediterranean region: Health and development profile

#### Socio-economic characteristics

This study includes the following 20 countries of EMR: Afghanistan, Bahrain, Djibouti, Egypt, Iran, Iraq, Jordan, Kuwait, Lebanon, Libya, Morocco, Oman, Pakistan, Qatar, Saudi Arabia, Sudan, Syria, Tunisia, United Arab Emirates (UAE) and the Yemen Republic. Somalia and Palestine (West Bank and Gaza) were excluded due to paucity of data in the required variables.

The region comprises a highly diverse group of countries with different socio-economic and health systems characteristics as shown in Table 1.

**Table 1:** Selected Socioeconomic, Health, and Healthcare System Financing Indicators for the Study Countries

Demographics						Health Indicators			Healthcare System Financing		
Country	Total Population (in thousands) (2015)	Population proportion under 15 Years (%) (2015)	Population proportion over 60 (%) (2015)	GNI per capita, PPP (current international $) (2016)	Human Development Index (HDI) (2015)	Life Expectancy (Years) (2015)	Infant Mortality (2015)	Total Health Expenditure (THE) as % of Gross Domestic Product (GDP) (2014)	General Government Health Expenditure (GGHE) as % of Gross Domestic Product (GDP) (2014)	Out of Pocket Expenditure (OOPS) as % of Total Health Expenditure (THE) (2014)	Skilled health professionals density (per 10,000 population)
Afghanistan	32,527	44	4	1900	0.479	60.5	66.3	8.2	2.9	64	7.7
Bahrain	1377	21.5	3.9	44,690^a^	0.823	76.9	5.3	5.0	3.2	23	32.9
Djibouti	888	32.7	6.3	2200^a^	0.470	63.5	54.2	10.6	6.8	36	10.3
Egypt	91,508	33.2	7.9	11,110	0.688	70.9	20.3	5.6	2.2	56	63.5
Iran	79,109	23.6	8.2	17,370^a^	0.774	75.5	13.4	7.5	3.7	41	23
Iraq	36,423	41	5	17,240	0.649	68.9	26.5	5.5	3.3	40	6.1
Jordan	7595	35.5	5.4	8980	0.741	74.1	15.4	7.5	5.2	21	66.1
Kuwait	3892	22.3	3.4	83,420^a^	0.799	74.7	7.3	3.0	2.6	13	63.4
Lebanon	5851	24	11.5	13,860	0.763	74.9	7.1	6.4	3.0	36	59.2
Libya	6278	29.8	7	11210^a^	0.719	72.7	11.4	5.0	3.7	26	87
Morocco	34,378	27.2	9.6	7700	0.645	74.3	23.7	5.9	2.0	58	15.1
Oman	4491	20.5	4.4	41,320^a^	0.795	76.6	9.9	3.6	3.2	6	78.1
Pakistan	188,925	35	6.6	5580	0.548	66.4	65.8	2.6	0.9	56	14
Qatar	2235	15.5	2.3	124,740^a^	0.855	78.2	6.8	2.2	1.9	7	196.1
Saudi Arabia	31,540	28.6	5	55,760	0.845	74.5	12.5	4.7	3.5	14	73.6
Sudan	40,235	40.5	5.2	4290	0.488	64.1	47.6	8.4	1.8	76	11.2
Syrian	18,502	37.1	6.4	N.A	0.553	64.5	11.1	3.3	1.5	54	33.2
Tunisia	11,254	23.4	11.7	11,150	0.723	75.3	12.1	7.0	4.0	38	45
UAE	9157	13.9	2.3	72,850	0.836	77.1	5.9	3.6	2.6	18	56.9
Yemen	26,832	40.3	4.7	2490	0.499	65.7	33.8	5.6	1.3	76	8.7

*N.A* not available

^a^Replaced by the most recent available data; N.B.: The most recent available data was used in representing the Eastern Mediterranean Region health and development profile

Population size ranges from approximately 1.38 million (Bahrain) to over 188 million (Pakistan) for 2015. The population in the region is generally young. The proportion of elderly population (60+ years) is on average 6.1%, ranging from 2.3% (UAE) to 11.7% (Tunisia).

There is a glaring inequality in income across countries. In 2016, gross national income (GNI) per capita in purchasing power parity (PPP) (current international $) ranges from as low as 1900 (Afghanistan) to around 125,000 (Qatar). According to the 2017 World Bank’s classification of countries by income level, only Afghanistan falls under the low income countries (LICs) group. Djibouti, Egypt, Jordan, Morocco, Pakistan, Sudan, Syria, Tunisia, and Yemen are classified as lower-middle income countries (LMICs); while Iran, Iraq, Lebanon, Libya fall under the upper-middle income countries (UMICs) group. The high income countries (HICs) group consists of Bahrain, Kuwait, Oman, Qatar, Saudi Arabia, and the United Arab Emirates.

In 2015, four countries in the region were categorized as very high on the Human Development Index (HDI ≥ 0.8), six countries as a high (with HDI between 0.7–0.799). Sudan, Djibouti and Afghanistan were classified as low human development countries (HDI ≤ 0.550) [24].

A number of countries in the region are in turmoil with Afghanistan, Iraq, Libya, Syria and Yemen being hit by war and active conflicts. The negative consequences of the wars (including the refugee crisis) on health and health systems in the region are immense. Attacks against health facilities, civilians, and providers are disrupting the provision and access to healthcare [25, 26]. In Libya, trauma centers and obstetric care are difficult to access in some conflict areas [26]. The wide spread of refugees in the region is leading to overload of the existing healthcare systems and present a major challenge for the host country and has several negative impacts on quality of healthcare services provided [27]. According to the United Nations High Commissioner for Refugees (UNHCR), more than 4.8 million registered Syrian refugees are displaced within countries in the EMR and Turkey [28]. Lebanon has over one million registered Syrian refugees, Jordan has over 656,000, Iraq around 250,000 and Egypt around 115,000 [28]. The wide spread of refugees in the region is leading to shortages in childhood vaccinations, drugs and access to clean water, food supplies, and sanitary housing [28]. Overcrowding and unsanitary living conditions for refugees is leading to the spread of infectious diseases [29–32].

#### Burden of disease

In 2015, life expectancy (LE) in the region ranged between 60.5 years (Afghanistan) to as high as 78.2 years for Qatar (Table 1). Despite the fact that some countries in the region made improvements in reducing childhood mortality, the progress in many countries fell short of what is required to achieve the Millennium Development Goal (MDG) 4 target of reducing under-5 mortality rate by two-thirds between 1990 and 2015 [1]. Non-communicable diseases (NCDs) and injuries in the region impose a heavy burden on health and health resources [1]. A notable increase in diabetes, cardiovascular diseases, cancer, and mental disorders has been noticed in the region [1–3]. Some countries in the region also carry a heavy burden of disease from communicable diseases. For example, in Djibouti the healthcare system faces an HIV epidemic, in addition to diarrheal diseases (including cholera and pneumonia) [33]. In Egypt, Hepatitis B and C continue to be a public health problem [34]. Jordan also suffers from Hepatitis in addition to respiratory infections and diarrheal diseases [35]. Currently, measles, poliomyelitis, leishmaniasis, and multidrug-resistant tuberculosis are among reemerging infections in the region [32].

#### Health system characteristics

General Government Health Expenditure (GGHE) as % of Gross Domestic Product (GDP) for 2014 ranged from as low as 0.9% for Pakistan to 6.8% for Djibouti. The out of pocket expenditure (OOPS) as % of total expenditure on health (TEH) is notably high with 15 out of the 20 countries having an OOPS greater than 20% of total health spending which is a threshold below which the risk of catastrophic spending is generally small (Table 1).

Only 7 out of the 20 countries in this study do not meet the minimum threshold of 22.8 skilled health professionals (doctors, nurses and midwives) per 10,000 population that was established in 2006 by the WHO to deliver the most basic health coverage [36].

## Methods

### Input-output data and data sources

Productivity is essentially concerned with output-input comparisons and is defined as the level of output per unit of input. Productivity can be used for comparing the performance of decision making units (DMUs) (specific country health system or health systems of the region) at a point in time. Productivity change refers to movements (increase/decrease) in the productivity of decision making units (DMUs) over a period of time (e.g. two time periods, *t* and *t + 1*) [37].

In this study, data on inputs and outputs were extracted from the WHO’s Global Health Observatory and Global Health Expenditure databases. Additional health and development data was obtained from the World Bank and UNDP reports/databases.

Following other studies on performance of health systems [15–17], total expenditure on health per capita at purchasing power parity (PPP) was used as a measure of input. The total expenditure on health per capita represents all the resources of the health system (current expenditure spent on all the factors of production and capital formation).

Output was represented by life expectancy at birth for both sexes and infant mortality rate (IMR) [16, 17]. The panel data on input and outputs covered the period between 2003 and 2014 inclusive (12 years).

The outputs measures were scaled into indices between 0 and 1 to create natural zeros and aspirational targets in line with the method used in calculating the human development index. The human development report sets the natural zero for life expectancy at 20 years based on historical evidence that no country in the twentieth Century had a life expectancy of less than 20 years. The aspirational target (maximum) is set at 85 years. The life expectancy index (LEI) for each country is thus calculated as [24]:

1 $$ LEI=\frac{Actual\kern0.5em value-\kern0.5em Minimum\kern0.5em value}{Maximum\kern0.5em value- Minimum\kern0.5em value} $$

For the negative output indicator, infant mortality rate, where more is bad, scaling was done so that a country with the lowest IMR will receive a higher score and the one with the highest IMR will receive a lower score. The Maximum and minimum values were set respectively as 96 and 1.5 based on global IMR values for 2015 as provided in Human Development Report 2016 [24]. To create the IMR index (IMRI), the country IMR figures were scaled as follows [38]:

2 $$ IMR=\frac{Maximum\kern0.5em value-\kern0.5em Actual}{Maximum\kern0.5em value- Minimum\kern0.5em value} $$

### Measuring productivity change

When measuring productivity change and similar to other empirical oriented methods, a small number of DMUs may risk the problem of degrees of freedom. To address this problem, Cooper et al. (2011) suggested the following rule of thumb: *n ≥* Max {*m* x *s*, 3(*m* + *s*)}, where *n* = number of DMUs (countries), *m* = number of inputs, *n* = number of outputs. Accordingly, the number of countries included in this study should be greater than 9 [39]. The number of countries, which is 20, is more than twice the expected minimum number. Furthermore, a period of 14 years is sufficient enough to register productivity changes.

In a situation where DMUs produce multiple outputs using multiple inputs, productivity change is measured using total factor productivity (TFP) index, which is also called multifactor productivity index (MFP). There are various approaches of measuring the TFP index. Regardless of the approach, a TFP index should satisfy the following requirements [37]:

i. In a scenario where a decision making unit produces the same output in time periods *t* and *t + 1*, and the input use is reduced by a certain percentage, then TFP should increase accordingly.
ii. Similarly, when outputs are increased between time periods *t* and *t + 1* by a certain percentage keeping the input use the same, TFP should also increase.

Output-oriented Malmquist total factor productivity index (MTFP) is used in computing productivity change over the period 2003 to 2014 in the 20 countries of the Eastern Mediterranean region. The index, which was introduced by Caves, Christensen and Diewart (1982), measures total factor productivity (TFP) change between two time points in terms of ratios of distance functions [40].

The concept of distance function, which is related to production frontiers, was first introduced by Malmquist (1953) and Shephard (1953) [35]. Distance function help to describe multiple input, multiple output production technology without a priori behavioral assumption (such as cost minimization). Distance function may have an input or output orientation. An output distance function, which is the orientation of this study, considers a maximal proportional expansion of the output vector given an input vector.

The MTFP between two time periods (*t* and *t + 1*) using period *t* and period *t + 1* technologies respectively is given as:

3 $$ {M}^t=\frac{D^t\left({x}^{t+1},{y}^{t+1}\right)}{D^t\left({x}^t,{y}^t\right)} $$

4 $$ {M}^{t+1}=\frac{D^{t+1}\left({x}^{t+1},{y}^{t+1}\right)}{D^{t+1}\left({x}^t,{y}^t\right)} $$

Where:

*M*^*t*^, *M*^*t* + 1^ – denote the MTFP in period *t* and *t + 1* respectively;

*D*^*t*^(*x*^1 + 1^, *y*^*t* + 1^) – refers to the output distance function which evaluates period *t + 1* data relative to the technology in period *t*;

*D*^*t*^(*x*^*t*^, *y*^*t*^) – output distance function evaluating period *t* data relative to technology in period *t*;

*D*^*t* + 1^(*x*^*t* + 1^, *y*^*t* + 1^) – output distance function evaluating period *t + 1* data relative to technology in period *t + 1*;

*D*^*t* + 1^(*x*^*t*^, *y*^*t*^) – output distance function evaluating period *t* data relative to technology in period *t + 1*;

Since there are two possible indices measured using period *t* and period *t + 1* technologies, the MTFP is defined as the geometric mean of the Eqs. 1 and 2 as follows:

5 $$ M={\left[\frac{D^t\left({x}^{t+1},{y}^{t+1}\right)\;{D}^{t+1}\left({x}^{t+1},{y}^{t+1}\right)}{D^t\left({x}^t,{y}^t\right)\kern1em {D}^{t+1}\left({x}^t,{y}^t\right)}\right]}^{\raisebox{1ex}{$1$}\!\left/ \!\raisebox{-1ex}{$2$}\right.} $$

Färe et al. further decomposed the MTFP in Eq. 3 into efficiency change (EFFCH) and technical/technological (TECHCH) change as follows [41]:

6 $$ M=\frac{D^{t+1}\left({x}^{t+1},{y}^{t+1}\right)}{D^t\left({x}^t,{y}^t\right)}\kern0.5em {\left[\frac{D^t\left({x}^{t+1},{y}^{t+1}\right)}{D^{t+1}\left({x}^{t+1},{y}^{t+1}\right)}\times \frac{D^t\left({x}^t,{y}^t\right)}{D^{t+1}\left({x}^t,{y}^t\right)}\right]}^{\raisebox{1ex}{$1$}\!\left/ \!\raisebox{-1ex}{$2$}\right.} $$

That is: M = TEFFCH × TECHCH.

The term outside the square brackets captures efficiency change and denotes whether the observation got closer or farther from the frontier over time. It is the ratio of the output-oriented technical efficiency between periods *t* and *t + 1*. The geometric mean of the two ratios inside the square brackets captures a shift in technology (frontier) or technical change.

MTFP index greater than 1 indicates growth in productivity, whereas a value less than 1 indicates a decline in productivity between periods *t* and *t + 1*. A value of 1 denotes stagnation in productivity.

There are a number of different methods to calculate the MTFP index. However, the most preferred method is one that uses data envelopment analysis (DEA)-like linear programming techniques. Four linear programming problems are solved for each decision making unit to estimate four distance functions under a constant returns to scale technology [37]. The technical efficiency (TE) change can further be decomposed into pure efficiency change (PECH) component and scale efficiency change (SECH) component by solving two additional linear programming problems under variable returns to scale technology [37]. Total factor productivity change is thus the product of technical change, pure technical efficiency change and scale efficiency change.

### Data analysis

DEA-based Malmquist Total Factor Productivity Index was estimated using data envelopment analysis computer program, DEAP version 2.1 [42]. To assess the robustness of the MPIs, bootstrapping was performed using STATA 12 statistical software. Further descriptive analysis was also conducted using MS Excel and Stata 12 statistical software.

## Results

### Descriptive analysis

Table 2 presents summary statistics of the output and input variables for the period covered, 2003–2014.

**Table 2:** Summary statistics - output/input variables, 2003–2014

Year	Output/input variable	Mean	SD	Min	Max
2003	LE (Output 1)	69.3	6.1	56.7	76.5
	IMR (Output 2)	33.1	26.1	8.9	89
	TEH per capita (Input)	851	998	67	3963
2004	LE	69.6	6.1	57	76.6
	IMR	32.1	25.5	8.7	86.7
	TEH per capita	831	952	88	3846
2005	LE	69.8	6.0	57.3	76.6
	IMR	31.0	24.9	8.4	84.4
	TEH per capita	774	806	88	3121
2006	LE	69.9	6.0	57.3	76.6
	IMR	30.1	24.4	8.2	82.3
	TEH per capita	780	772	87	2940
2007	LE	70.2	5.9	57.5	76.7
	IMR	29.1	23.9	8	80.4
	TEH per capita	795	733	89	2685
2008	LE	70.6	5.7	58.1	76.8
	IMR	28.2	23.4	7.8	78.6
	TEH per capita	767	653	111	2212
2009	LE	70.9	5.6	58.6	77
	IMR	27.4	22.9	7.6	76.8
	TEH per capita	1009	923	123	3053
2010	LE	71.1	5.5	58.8	77.3
	IMR	26.5	22.5	7.1	75.1
	TEH per capita	908	748	127	2583
2011	LE	71.2	5.4	59.2	77.5
	IMR	25.8	22.0	6.7	73.4
	TEH per capita	891	739	130	2582
2012	LE	71	5.7	59.5	77.8
	IMR	25	21.5	6.3	71.7
	TEH per capita	972	782	123	2856
2013	LE	71.2	5.6	59.9	77.9
	IMR	24.2	21.0	5.9	69.9
	TEH per capita	1003	822	127	2917
2014	LE	71.2	5.6	59.9	78.1
	IMR	23.5	20.5	5.6	68.1
	TEH per capita	1093	918	129	3071

*LE* life expectancy at birth, *IMR* infant mortality rate per 1000 live births, *TEH* total expenditure on health

As can be seen from Table 2, there has been a notable gap between the minimum and maximum values of the output/input variables, and the gap tend to show a slight decrease over the years. The gap in life expectancy, which was 19.8 years in 2003, decreased marginally to 18.2 years in 2014. Similarly the gap in IMR declined to 62.5 points per 1000 live births in 2014 compared to 80.1 in 2003. The difference in TEH per capita was PPP$ 3896 in 2003. This was reduced to PPP$ 2942 in 2014. The mean life expectancy at birth that was 69.3 in 2003 increased by a mere 1.9 years over the period of 12 years. Similarly, the mean IMR declined from 33.1 per 1000 live births in 2003 to 23.5 in 2014; and TEH per capita increased from PPP$ 851 to 1093.

Table 3 shows the change in the study input/output from 2003 to 2014 by country.

**Table 3:** Input/output of Health Systems in the Eastern Mediterranean Region

Country	Output						Input		
	LE 2003	LE 2014	AAR of change (%)	IMR 2003	IMR 2014	AAR of change (%)	TEH 2003	TEH 2014	AAR of change (%)
Afghanistan	56.7	59.9	0.5	89	68.1	−2.4	89	167	5.89
Bahrain	75	76.8	0.22	9.9	5.6	−5.05	1398	2273	4.52
Djibouti	58	63	0.75	74.6	55.8	−2.61	113	338	10.47
Egypt	68.6	70.8	0.29	31.9	21	−3.73	369	594	4.42
Iran	70.5	75.4	0.61	24.3	13.9	−4.95	610	1298	7.11
Iraq	66.5	67.9	0.19	34.1	27.2	−2.03	67	667	23.23
Jordan	72.3	74	0.21	21.5	15.8	−2.76	653	798	1.84
Kuwait	73.4	74.6	0.15	10.4	7.7	−2.7	2257	2320	0.25
Lebanon	73.5	74.8	0.16	14.1	7.3	−5.81	919	987	0.65
Libya	71.3	72.4	0.14	21.9	11.9	−5.39	847	806	−0.45
Morocco	69.9	74.1	0.53	37.4	24.6	−3.74	225	447	6.44
Oman	73.6	76.4	0.34	11.9	10	−1.57	1120	1442	2.32
Pakistan	63.5	66.2	0.38	82.9	67.4	−1.86	86	129	3.43
Qatar	76.5	78.1	0.19	9.5	7	−2.74	3963	3071	−2.29
Saudi Arabia	73	74.4	0.17	17.6	12.9	−2.78	1184	2466	6.9
Sudan	59.6	63.8	0.62	63	48.8	−2.3	101	282	9.78
Syria	73	64.4	−1.13	17.5	11.7	−3.59	187	376	9.56
Tunisia	73.7	75.1	0.17	22	12.6	−4.94	360	785	7.34
United Arab Emirates	74.9	76.9	0.24	8.9	6.1	−3.38	2283	2405	0.47
Yemen, Republic	61.9	65.4	0.5	60.1	35.1	−4.77	197	202	0.23

Over the twelve-year period, almost all countries had an increase in TEH per capita with a positive average annual rate (AAR) of change ranging from 0.25% (Kuwait) to 23.23% (Iraq) except for Qatar (AAR_THE_ -2.29) and Libya (AAR_THE_ -0.45). Life expectancy increased for all countries except for Syria with a decrease of approximately 9 years. This decline in life expectancy in Syria could probably be attributed to the effects of war on the country. During the same period, Djibouti was able to increase its LE from 58 years to 63 years with an AAR_LE_ increase of 0.75% or 5 years. IMR was reduced during the same period, with Lebanon achieving the highest annual reduction rate of − 5.81%.

### Technical efficiency scores

Table 4 shows the mean output-based DEA technical efficiency scores for the period 2003–2014. These are produced using output distance functions evaluating each year’s output-input data with respect to the same year’s technology (Additional file 1).

**Table 4:** Mean technical efficiency scores 2003–2014

Country	Mean CRS TE	Mean VRS TE	Mean Scale efficiency
Afghanistan	0.806	0.881	0.915
Bahrain	0.127	1.000	0.127
Djibouti	0.558	0.767	0.728
Egypt, Arab Republic	0.337	0.919	0.367
Iran	0.179	0.960	0.186
Iraq	0.677	0.956	0.708
Jordan	0.215	0.965	0.223
Kuwait	0.091	0.979	0.093
Lebanon	0.180	0.994	0.181
Libya	0.232	0.956	0.243
Morocco	0.487	0.965	0.505
Oman	0.169	0.998	0.169
Pakistan	0.930	0.967	0.962
Qatar	0.067	1.000	0.067
Saudi Arabia	0.113	0.954	0.119
Sudan	0.567	0.806	0.704
Syrian Arab Republic	0.889	1.000	0.889
Tunisia	0.301	1.000	0.301
United Arab Emirates	0.090	0.999	0.090
Yemen, Republic	0.641	0.841	0.762
Overall mean	0.383	0.945	0.405

*CRS* constant returns to scale, *TE* technical efficiency, *VRS* variable returns to scale

The overall mean CRS technical efficiency score of 0.383 indicates that on average, there is inefficiency of about 62%. The mean technical efficiency score is, however, closer to the frontier (i.e. TE score = 1) when the scale efficiency effect is removed by estimating a VRS model. Table 4 further indicates that there is a wide variation in the mean CRS TE among the different countries in the region. Interestingly, it is observed that the high income of the group have mean efficiency scores that are less than 20%, which is due to high levels of scale inefficiency.

### Malmquist Total factor productivity change

Results from the decomposition of the Malmquist index summary of country means for the study period are reported in Table 5. The bootstrap estimates of the means are also presented in Table 6. The bootstrap estimates are robust and statically significant (*p* < 0.01). Under Table 5, the estimates are reported in five columns, technical efficiency change (EFFCH) which is decomposed further into pure efficiency change (PECH) and scale efficiency change (SECH) respectively and technical or technological change (TECHCH). When analyzing the temporal change in productivity between 2003 and 2014 using the MTFP index, a value of the MTFP index or any of its components less than one denotes that the productivity of the DMU is regressing over the time period; whereas values greater than one denote improvement in productivity; and a value of one denotes stagnation. In all but five countries covered in this study, decline in the mean total factor productivity was observed during the period 2003–2014. Three countries – Libya, Qatar and Yemen had marginal growth in total factor productivity ranging between 1.2% (Yemen) and 2.7% (Qatar). Kuwait and Lebanon exhibited stagnation, i.e. no change in total factor productivity.

**Table 5:** Malmquist Index summary of country means, 2003–2014

Country	TEFFCH	TECHCH	PECH	SECH	MTFPCH
Afghanistan	1.019	0.943	1.01	1.009	0.96
Bahrain	1.064	0.903	1	1.064	0.961
Djibouti	0.995	0.927	1.008	0.987	0.923
Egypt	1.074	0.898	1.006	1.068	0.965
Iran	1.047	0.903	1.005	1.041	0.945
Iraq	0.907	0.904	0.987	0.919	0.82
Jordan	1.094	0.902	1.001	1.093	0.987
Kuwait	1.108	0.903	0.999	1.108	1
Lebanon	1.108	0.903	1.001	1.107	1
Libya	1.125	0.902	1.004	1.121	1.015
Morocco	1.047	0.906	1.006	1.041	0.949
Oman	1.084	0.904	1	1.084	0.98
Pakistan	1.03	0.945	1.009	1.021	0.973
Qatar	1.136	0.904	1	1.136	1.027
Saudi Arabia	1.041	0.903	0.998	1.044	0.94
Sudan	1.007	0.917	1.008	0.999	0.924
Syria	1.046	0.896	1	1.046	0.937
Tunisia	1.043	0.9	1	1.043	0.938
United Arab Emirates	1.105	0.903	1	1.105	0.998
Yemen, Republic	1.115	0.908	1.024	1.088	1.012
Mean	1.058	0.909	1.003	1.055	0.962

*TEFFCH* technical efficiency change, *TECHCH* technological change, *PECH* pure efficiency change, *SECH* scale efficiency change, *MTFPCH* Malmquist total factor productivity change index

**Table 6:** Bootstrapped means of Malmquist index

	TFPCH	TEFFCH	TECHCH	PECH	SECH
Afghanistan	1.047	0.996	1.069	0.998	0.995
	(0.032)	(0.056)	(0.054)	(0.041)	(0.026)
Bahrain	1.046	0.971	1.143	1.000	0.971
	(0.040)	(0.091)	(0.132)	(0.000)	(0.091)
Djibouti	1.087	1.017	1.090	0.993	1.024
	(0.026)	(0.052)	(0.057)	(0.010)	(0.049)
Egypt	1.037	0.956	1.146	0.995	0.962
	(0.010)	(0.066)	(0.100)	(0.002)	(0.067)
Iran	1.063	0.988	1.142	0.995	0.992
	(0.035)	(0.082)	(0.099)	(0.002)	(0.081)
Iraq	1.339	1.195	1.139	1.014	1.167
	(0.212)	(0.189)	(0.106)	(0.010)	(0.167)
Jordan	1.016	0.939	1.143	0.999	0.940
	(0.028)	(0.068)	(0.108)	(0.002)	(0.067)
Kuwait	1.027	0.950	1.143	1.001	0.950
	(0.084)	(0.094)	(0.104)	(0.000)	(0.094)
Lebanon	1.001	0.926	1.144	0.999	0.928
	(0.016)	(0.063)	(0.108)	(0.001)	(0.063)
Libya	1.034	0.971	1.144	0.996	0.972
	(0.097)	(0.121)	(0.101)	(0.002)	(0.119)
Morocco	1.063	0.980	1.131	0.994	0.987
	(0.043)	(0.064)	(0.082)	(0.002)	(0.065)
Oman	1.033	0.957	1.142	1.000	0.957
	(0.047)	(0.067)	(0.104)	(0.001)	(0.066)
Pakistan	1.031	0.978	1.067	0.992	0.983
	(0.025)	(0.034)	(0.047)	(0.015)	(0.025)
Qatar	0.985	0.907	1.142	1.000	0.907
	(0.034)	(0.064)	(0.116)	(0.000)	(0.064)
Saudi Arabia	1.073	0.994	1.143	1.002	0.991
	(0.044)	(0.070)	(0.093)	(0.001)	(0.069)
Sudan	1.091	1.010	1.108	0.993	1.014
	(0.047)	(0.065)	(0.074)	(0.017)	(0.057)
Syria	1.085	0.983	1.155	1.000	0.983
	(0.073)	(0.074)	(0.120)	(0.000)	(0.074)
Tunisia	1.067	0.981	1.146	1.000	0.981
	(0.012)	(0.060)	(0.096)	(0.000)	(0.060)
United Arab Emirates	1.005	0.927	1.143	1.000	0.927
	(0.032)	(0.048)	(0.087)	(0.000)	(0.048)
Yemen, Republic	0.992	0.917	1.122	0.977	0.941
	(0.022)	(0.041)	(0.063)	(0.008)	(0.047)

Coefficients are means of Malpquist indices over time and across DMUs. Robust standard errors in parentheses, *p* < 0.01

As can be inferred from Table 5, the decline in total factor productivity over the years was driven by regress in technology. It is observed that in all countries, the technical change component of the Malmquist TFP index is less than unity (range: 0.896 to 0.945). Out of five countries that registered efficiency change of more than 10% (range: 10.5 to 13.6), three were high income countries of the Gulf Cooperation Council (Kuwait, Qatar and United Arab Emirates). Djibouti and Iraq, which are regarded as lower- and upper-middle income countries respectively had a decline in efficiency over the years. It is also seen that the change in efficiency was mainly driven by change in scale efficiency. Overall, total factor productivity declined in the region by 3.8%. This was due to a 9.1% decline in technical change, which overshadowed the 5.8% increase in efficiency.

The yearly TFP indices for each country demonstrate that not all the years showed a decline in total factor productivity. Table 7 presents information on the number of years (out of 11) that the total factor productivity change and its components registered growth, stagnation or decline.

**Table 7:** Number of years of growth, stagnation or decline of Malmquist total factor productivity change index, 2003–2014

Country	Number of years MTFPCH index		
	> 1	=1	< 1
Afghanistan	5	0	6
Bahrain	5	0	6
Djibouti	1	0	10
Egypt	2	0	9
Iran	3	0	8
Iraq	4	0	7
Jordan	4	0	7
Kuwait	8	0	3
Lebanon	7	0	4
Libya	9	0	2
Morocco	1	1	9
Oman	7	0	4
Pakistan	4	0	7
Qatar	7	0	4
Saudi Arabia	3	0	8
Sudan	3	0	8
Syria	6	0	5
Tunisia	1	0	10
United Arab Emirates	6	0	5
Yemen, Republic	7	0	5

In three of the 20 countries (Djibouti, Morocco and Tunisia) there was only 1 year of total factor productivity growth. On the other hand, Libya and Kuwait had 9 years and 8 years of total factor productivity growth respectively.

**Fig. 1:**
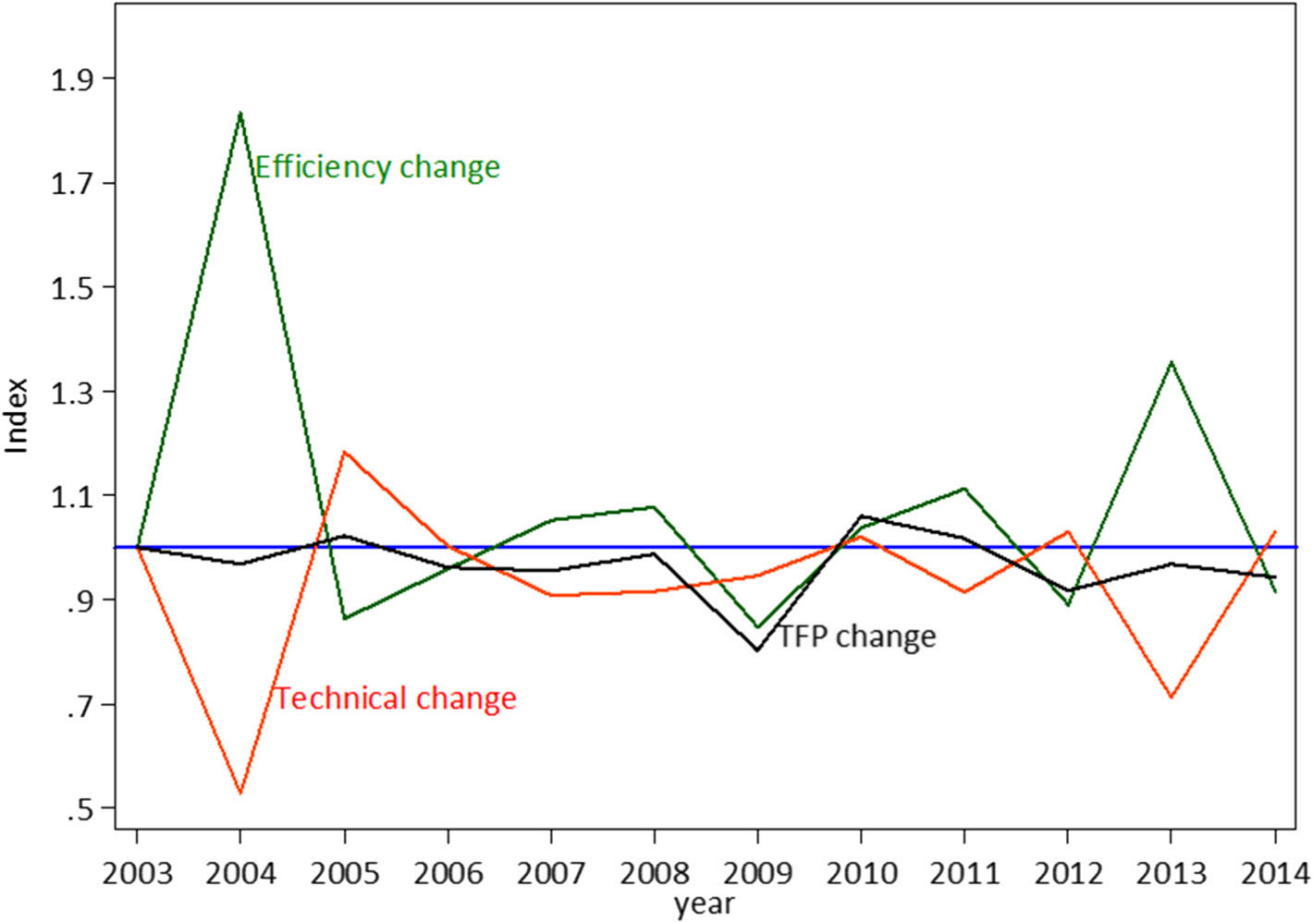
Malmquist Index summary of annual means, 2003–2014, all countries of EMR

Figure 1 portrays the overall summary of annual means of TFPCH and its components for the region overall.

The pattern in Fig. 1 shows that efficiency change and technical change components of the Malmquist index moved in opposite directions thus diluting growth in total productivity. Moreover, it is observed that the TFP change was always below 1 except in 2005 and 2010.

## Discussion

The assessment of health system productivity change over time which comprises efficiency change and technical change is challenging due to limited availability of input and output data over time and across country [43].

Life expectancy has increased by more than 1 year (1.9 years) on average since 2003 for countries in the region. The mean infant mortality rate also declined to 23.5 per 1000 live births in 2014. Despite improvements in LE and IMR, and an increase in inputs, TEH per capita over the study period, there is inefficiency of about 62% in the region (Table 4). The study points to the existence of widespread inefficiency over the years. Thus, there is a tremendous opportunity to expand outputs given the level of resources. Only three countries – Afghanistan, Pakistan and Syria – had a mean CRS technical efficiency score exceeding 0.8, which is also below the efficient frontier.

Ninety percent (18/20 countries) of the DMUs experienced an increase in technical efficiency, however all countries in this study experienced a regress in technology. The obvious technology regress between 2003 and 2014 could be attributed to either a low adoption of new technologies within the region and/or to technical problems faced by using outdated technologies. Furthermore, the productivity measures have indicated that, while on average there was close to 6% growth in efficiency, technical change declined by an average of 9% over the study period. The mean technical change for all the countries included was less than one, implying that the technology (production) frontier shifted downwards. The 6% change in efficiency indicates a catch-up growth that was outpaced by technical regress leading to decline in productivity. A point of note is that, the catch-up growth for two countries (Djibouti and Iraq) was less than one, making them the only two countries with a downward shift in the production frontier and efficiency change.

Possible sources for inefficiencies in healthcare systems in the region vary from suboptimal mix between public and private healthcare financing, and heavy reliance on external sources of funding, to suboptimal provision of primary care, to high dependence on expatriate healthcare workforce, to inefficient utilization of hospital beds, generic drugs, technology, IT systems or electronic medical records, to fraud, corruption, and others.

Inadequate financing of basic healthcare services constitute a major problem for Afghanistan who relies heavily on external sources of funding [23], while in Saudi Arabia, healthcare funding through the government budget is highly linked to oil revenues. Fluctuations in oil revenues will highly affect the level of input/resources available for health [44].

A number of countries in the region including Bahrain, Iran, Kuwait, Lebanon, Sudan, and Yemen are working to optimize the allocation of resources for primary care services and strengthen quality primary care services [45–49]. In Bahrain, a major source of inefficiency is the overutilization of hospital beds with patients that could have been treated at the primary healthcare level [50]. Iran through its extended primary care network in rural areas was able to improve on health indicators and efficiency of its healthcare system [45]. Kuwait is trying to electronically connect primary and family healthcare centers with secondary and tertiary centers for better patient referral system [46]. The Ministry of Public Health in Lebanon, is expanding its primary care network by adding 50 new primary healthcare centers each year [47]. Sudan through its primary health care expansion plan (2012–2016) is aiming to cover the whole population with quality primary care services [48].

Healthcare workforce specifically in the Gulf Cooperation Council Countries relies heavily on expatriate workers [44, 46, 50]. Recruiting, proving the appropriate training, and retaining quality healthcare workforce impose a major challenge to these healthcare systems. Kuwait, Saudi Arabia and the UAE are aiming to train national healthcare workforce to overcome this problem.

Another probable source of inefficiency in some countries in the region is the overutilization of prescription drugs. Prescription medicines utilization should be monitored as the cost of these drugs constitutes a large portion of healthcare spending. Proper utilization and management policies like the introduction of pharmacy benefit management (PBM), the use of unified electronic medical records, use of cost-effective medications, the use of generic vs. brand medications, the timely initiation of essential medication therapy, and proper follow up to ensure adherence to that therapy could help in curbing cost and increasing efficiency of the system. For example, 90% of the pharmaceuticals consumed in Egypt are produced locally. However, because medications are highly subsidized by the Egyptian government this leads to their overuse [34]. Finally, there is a need for future research to examine and thoroughly analyze the sources of inefficiencies specific for each country.

### Limitations of the study

Despite that the outputs used in this study, i.e. life expectancy and infant mortality are imperfect indicators of efficiency and productivity of health spending because they do not reflect morbidity or the quality of life in the region, however, the variables are highly correlated with indicators of population health status and to date they remain to be the best available proxies of it. Indicators such as life expectancy and infant mortality capture generic information about population health and are often considered when assessing performance of health systems [15–17]. As in any other study on efficiency and productivity, the findings are sensitive to data errors. Countries at different stages of economic and social development have different reporting systems, which can affect consistency of data leading to errors in analysis and interpretation of findings. In the absence of robust vital registration systems and system of health accounts, data on outputs and inputs for some countries may be estimates, further compounding the problem.

## Conclusion

The findings of this study provide some information on productivity change, which comprises efficiency change and technical change using panel data of the national health systems of 20 Member Countries of the WHO Eastern Mediterranean region for the period 2003–2014.

It is evident that persistence of the level of inefficiency observed in the study countries will negatively affect progress towards achieving the targets of Sustainable Development Goal (SDG) 3, which is about ensuring healthy lives and promoting well-being for all at all ages [51]. Expanding and sustaining access to needed health services without financial hardship in line with the requirements of universal health coverage will be highly challenging, as this will lead to dwindling of resources for health. Health sector efficiency improvement is one of the ways to expand fiscal space for health [52].

Too often, healthcare policy debate focuses on cutting or generating more funds for healthcare while ignoring on ways to transform these funds into an efficient healthcare system. Improving health care systems productivity, while containing rapidly escalating healthcare costs, is a key policy challenge for countries in this region. One way to containing the growth of health care costs is through improving the efficiency of the health care system. Improving efficiency of the healthcare system will be important to meet rapidly growing health care demand, without putting the public finances of countries on an unsustainable financing path. A number of countries in the region are undergoing reform. However a “one-size-fits-all” approach to reform is not recommended. Each country should test and implement different approaches according to their needs to improve on the efficiency of their healthcare system.

## Additional file

Additional file 1: DEA Analysis. (TXT 27 kb)

## Abbreviations

CRS: Constant returns to scale
DEA: Data envelopment analysis
DMU: Decision making unit
EMR: Eastern Mediterranean Region
GDP: Gross domestic product
GGHE: General Government Health Expenditure
HIC: High Income Country
IMR: Infant mortality rate
LE: Life Expectancy at Birth (both sexes)
LEI: Life expectancy index
LMIC: Low middle income country
MDG 4: Millennium Development Goal 4
MTFP: Malmquist total factor productivity index
MTFPCH: Malmquist total factor productivity change index
NCD: Non-Communicable Diseases
OOPS: Out of pocket expenditure
PECH: Pure efficiency change
PPP: Purchasing power parity
SDG: Sustainable development goal
TE: Technical efficiency
TECH: Technical efficiency change
TECHCH: Technical or Technological Change
TEH: Total Expenditures on Health
UAE: United Arab Emirates
UMIC: Upper middle income country
VRS: Variable returns to scale
